# Metabolic dysfunction-associated steatotic liver disease and neurocognitive and reward circuit dysfunction: implications for mental disorders

**DOI:** 10.1017/S1092852926100984

**Published:** 2026-05-26

**Authors:** Nina Sachi Mollin, Gia Han Le, Christine E. Dri, Elim Yang, Folkert van Bruggen, Kangguang Lin, Stavroula Bargiota, Heidi K.Y. Lo, Roger Ho, Taeho Greg Rhee, Bing Cao, Angela T.H. Kwan, Roger S. McIntyre

**Affiliations:** 1 University of Toronto, Canada; 2 Brain and Cognition Discovery Foundation, Canada; 3 University of Toronto Institute of Medical Science, Canada; 4 University Medical Center Groningen Department of Primary and Long-term Care, Netherlands; 5 Department of Psychiatry and Behavioral Sciences, The Chinese University of Hong Kong Faculty of Medicine, Hong Kong; 6 Department of Psychiatry, University of Thessaly School of Health Sciences, Greece; 7 The University of Hong Kong Department of Psychiatry, Hong Kong; 8 National University of Singapore Yong Loo Lin School of Medicine, Singapore; 9 Psychiatry, Yale University School of Medicine, USA; 10 Southwest University, China; 11 Department of Pharmacology and Toxicology, University of Toronto, Canada; 12 Department of Psychiatry, University of Toronto, Canada

**Keywords:** Fatty liver, cognition, executive function, MASLD, MASH, anhedonia, reward

## Abstract

Metabolic dysfunction-associated steatotic liver disease (MASLD) (formerly known as nonalcoholic fatty liver disease) is the most prevalent liver disease globally. Emerging evidence suggests MASLD-related metabolic and inflammatory processes may disrupt frontostriatal–limbic circuits, contributing to alterations in cognitive and reward-related brain function. This systematic review aimed to examine whether MASLD is associated with changes in cognitive functioning and reward-related processes, and whether these changes correspond to structural and functional brain alterations. A systematic search of PubMed, Web of Science, and Google Scholar was conducted from inception to November 11, 2025, using terms related to MASLD, cognition, and reward. Eligible studies included adult MASLD populations and observational designs assessing cognitive and/or reward outcomes. Studies involving neuropsychiatric or neurological disorders or medications known to affect cognition were excluded. Fourteen studies (eight cross-sectional, four cohort or case–control, and two Mendelian randomization) found MASLD significantly associated with poorer attention, executive function and memory, often proportional to disease severity. Neuroimaging studies reported significant reductions in cortical thickness, hippocampal and white matter volume, increased white matter hyperintensities, and cerebrovascular dysfunction (*p* < 0.05), most pronounced in executive and visuospatial regions. Limited evidence suggested alterations in small-to-moderate circuits, including nucleus accumbens dysfunction and reduced hippocampal–orbitofrontal connectivity. MASLD is associated with cognitive impairment, reward-related neural systems, and structural and cerebrovascular brain alterations. These findings reinforce its characterization as a multisystem condition affecting brain health and neuropsychiatric risk. Longitudinal studies with standardized criteria and confounder adjustment are needed to clarify causal mechanisms.

## Introduction

Hepatic steatosis, commonly referred to as fatty liver, comprises a spectrum of disorders, including metabolic dysfunction-associated steatotic liver disease (MASLD; formerly known as nonalcoholic fatty liver disease [NAFLD]) and alcohol-associated fatty liver disease. In recent years, there has been growing interest in the potential implications of MASLD for brain health, particularly given its high global prevalence and multisystem consequences.^1^^,^^2^ Recent estimates suggest that MASLD affects approximately 25%–32% of the global population with higher estimates in persons with risk factors (eg obesity, type II diabetes mellitus [T2DM]), highlighting its substantial societal and healthcare burden.^1^^,^^2^

Beyond hepatic and cardiometabolic abnormalities, MASLD is increasingly conceptualized as a multisystem disorder characterized by metabolic dysfunction, systemic inflammation, insulin resistance (IR), and vascular abnormalities, processes plausibly linked to central nervous system structure and function.^1^^,^^3^ Specifically, the systemic disturbances may affect frontostriatal and limbic circuits, which are highly sensitive to metabolic and cerebrovascular stress and subserve executive control, memory, motivation, and reward processing.^1^^,^^3^ As such, MASLD has been associated with cognition and reward through shared metabolic, inflammatory, and vascular pathways affecting common neural substrates.^14^^,^^20^ Emerging evidence indicates that MASLD is associated with adverse neurological and psychiatric outcomes, including stroke, mild cognitive impairment (MCI), and mood disorders, which can substantially reduce the quality of life.^1^^–^^4^ However, it remains unclear whether these associations reflect direct effects of liver pathology, shared metabolic risk factors, or bidirectional relationships. Consistent with the mechanistic framework previously described, MASLD has been associated with worse performance on cognitive testing and reduced total brain volume.^5^ Neuroimaging findings suggest that the aforementioned structural alterations involve brain regions supporting higher-order cognitive and affective processes. Notably, brain regions vulnerable to volume loss in MASLD overlap with integral frontostriatal and limbic networks that subserve executive control, motivation and reward processing.^6^^–^^8^ The overlap between regions affected in MASLD and frontostriatal–limbic networks raises the possibility that liver-related metabolic and vascular changes may influence both cognitive performance and reward-related processing through shared neural substrates.

In this review, cognition and reward are considered related but distinct neurobehavioral domains, with cognition encompassing attention, memory, and executive control, and reward referring to motivation, reward valuation, and reinforcement learning, while acknowledging partial overlap in their underlying neural substrates. It is acknowledged that the mechanisms supporting cognitive processes (eg working memory) partially overlap with the neural substrates subserving reward-related behavior.

Despite limited research examining the relationship between liver disease and reward processing, available studies suggest that MASLD is associated with brain atrophy and related neuropsychiatric deficits.^9^ However, there remains a gap in the literature regarding how cognitive and reward system functioning vary across stages of steatosis and fibrosis.^9^ Specifically, it remains unclear whether increasing disease severity is associated with greater impairments in cognitive functioning and/or reward processing. It is also unknown whether alterations in reward-related neurocircuitry are directly influenced by liver pathology or arise secondary to comorbid psychiatric conditions, as individuals with severe mental illness (SMI) are disproportionately affected by MASLD and independently exhibit deficits in cognitive and reward-related functioning.^2^^,^^4^^,^^6^^,^^10^^,^^11^

Clarifying the aforementioned associations is essential for understanding the neurobiological consequences of MASLD and identifying potential targets for the prevention and treatment of cognitive and reward-related impairments. Guided by the premise that MASLD-related metabolic, inflammatory, and vascular processes may converge on frontostriatal and limbic systems, the present systematic review aims to comprehensively synthesize the existing literature examining the effects of MASLD on cognitive performance and/or reward system functioning. Given the predominance of cross-sectional evidence, this review does not infer causality but rather synthesizes observed associations.

## Methods

### Eligibility criteria

Eligible studies enrolled adult populations with a diagnosis of nonalcoholic liver fibrosis or steatosis (based on Fibrosis-4 [FIB-4] index, computed tomography [CT], magnetic resonance imaging [MRI], ultrasound (US), liver stiffness measurement). Exclusion criteria were applied to reduce confounding factors known to independently affect cognitive and reward-related functioning, with the specific aim of isolating associations attributable to MASLD-related pathology and enhancing internal validity. Studies were excluded if they were primarily designed to investigate neurodegenerative disorders or major psychiatric disorders (eg dementia, schizophrenia, or alcohol use disorder), or if cognitive or reward outcomes were the primary consequence of these disorders rather than MASLD.

Studies involving participants with diagnosed neuropsychiatric or neurological disorders affecting cognition, or those receiving medications known to substantially influence cognitive performance (eg antidepressants, antipsychotics, stimulants, benzodiazepines, and anticholinergics), were excluded to minimize competing sources of cognitive impairment and reduce the risk that observed deficits would reflect comorbid conditions rather than MASLD-specific effects. Participants with hepatitis C were not eligible.

Although studies explicitly focused on psychiatric or neurodegenerative disorders were excluded, the broader clinical relevance of psychiatric comorbidity in MASLD, such as SMI, is addressed in the interpretation and discussion of findings. Although this approach may limit generalizability given the high co-occurrence of MASLD and psychiatric illness; however, these exclusions were implemented to strengthen methodological transparency and permit interpretation directly attributable to MASLD-related neurocognitive associations. This approach was intended to improve internal validity while acknowledging that psychiatric comorbidity represents an important clinical feature of real-world MASLD populations and is considered in the interpretation of findings.

Included studies were required to assess cognitive and/or reward-related outcomes using validated neurocognitive measures (ie MCI, Montreal Cognitive Assessment [MoCA], Cognitive Change Index, Pattern Comparison Test, and Auditory Verbal Learning Test) and/or reward system assessments (ie Probabilistic Reward Test [PRT], Effort Expenditure for Rewards Task [EEfRT], Snaith–Hamilton Pleasure Scale (SHAPS), Temporal Experience of Pleasure Scale [TEPS], electroencephalogram [EEG], functional MRI [fMRI]). Furthermore, only original observational studies (eg cross-sectional, case–control design, and Mendelian randomization) published in English in peer-reviewed journals were considered.

### Search and selection strategy

**Figure 1.**
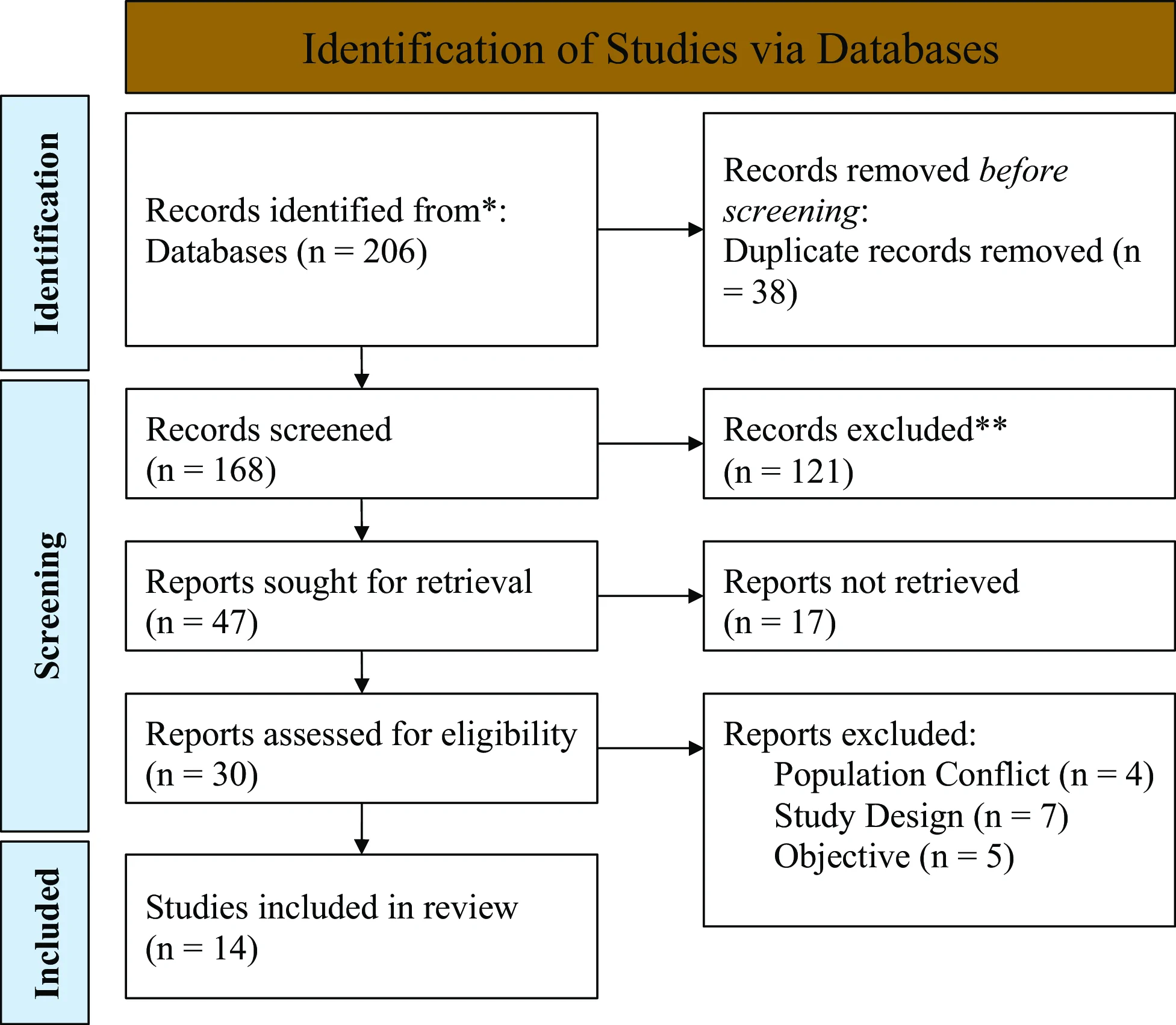
PRISMA flowchart of the study selection process. *Note:* PRISMA flow diagram outlining study identification, screening, eligibility, and inclusion. Searches were conducted in PubMed, Web of Science, and Google Scholar (inception—November 11, 2025), with an updated search on January 24, 2026. No additional eligible studies were identified. Figure 1. long description.

Following PRISMA guidelines, Figure 1 illustrates the literature search process conducted from inception to November 11, 2025, using online databases including PubMed, Web of Science, and Google Scholar.^12^ In addition, the reference lists of the studies and reviews obtained on similar topics were cross-checked and searched. The databases PubMed and Web of Science were searched using the following Boolean logic search string: (“Fatty Liver” OR “NAFLD” OR “MASLD” OR “Liver steatosis”) AND ((“Cognitive” OR “Cognition”) OR (“Reward system” OR “Reward” OR “Motivation”)). Limits were applied for the language of publication (ie English). Google Scholar was used to identify additional records, with the first 100 screened by relevance. The open-access software Covidence was used to screen articles. Two reviewers (NSM and EY) independently screened the titles and abstracts for eligibility, followed by a full-text review using the same criteria. Discrepancies were resolved through discussion and consensus, guided by prespecified eligibility criteria.

### Data collection process

We included studies that assessed cognitive and/or reward system functioning using validated measures. Cognitive outcomes included standardized neuropsychological tests assessing domains such as memory, attention, and executive function (eg MCI, MoCA, and PRT). Cognitive and reward outcomes were categorized as related but distinct domains to allow synthesis across heterogeneous measures while preserving differences in underlying neurobehavioral constructs. Findings were therefore summarized and interpreted separately within each domain, with cross-domain comparisons made only where studies explicitly assess both constructs. Reward system functioning was assessed using broad categories of measures, including behavioral tasks and self-reported questionnaire/survey measures, commonly used in reward and anhedonia literature. Behavioral tasks included PRT and EEfRT, while self-reported measures included questionnaires assessing the degree of anhedonia (eg SHAPS and TEPS). Neuroimaging and neurophysiological measures relevant to reward and cognitive circuitry, including PET, EEG, and fMRI, were also included when available. These measures are provided as representative examples rather than an exhaustive list.

### Methodological quality assessment

Methodological quality was assessed using the National Institutes of Health (NIH) Quality Assessment Tools.^13^ The tool appropriate to each study design (cross-sectional, cohort, and case–control) was selected from the NIH assessment framework.^13^ Each study was evaluated across tool-specific domains using “Yes,” “No,” “Cannot Determine,” or “Not Responded” responses. Design-specific domains were assessed with specific research questions, with an emphasis on confounding, methodological limitations, and the validity and reliability of exposure and outcome measurement. Overall study quality was categorized as good, fair, or poor based on the number and severity of methodological limitations. Studies were rated as “*good*” if most criteria were met with no major sources of bias, “*fair*” if some criteria were unmet or unclear without critical flaws, and “*poor*” if one or more critical domains were not met. Quality ratings were considered during data synthesis and interpretation to contextualize the strength and consistency of the reported associations. Specifically, findings from studies rated as “*fair*” or *“poor”* were interpreted with greater caution, and study quality was considered when evaluating the overall strength and coherence of evidence across domains. Two reviewers independently conducted all assessments, with disagreements resolved by consensus.

## Results

### Study selection

The initial search generated 206 results from PubMed, Web of Science and Google Scholar combined. After removing duplicates, 168 studies remained for the title and abstract screening. In the 168 studies, 14 were eligible for full-text screening, with the majority being excluded due to objective (ie the study was not relevant to the research question) or population conflicts (ie the subjects did not meet eligibility criteria) or study design (ie study design did not meet eligibility criteria). The full-text screening identified 14 eligible studies: 12 relevant to cognition and 2 to reward system functioning. The limited number of studies assessing reward-related outcomes reflects the emerging nature of this area and should be considered when interpreting findings related to reward circuitry. Consequently, conclusions regarding reward processing are necessarily more tentative than those concerning cognition, which are supported by a comparatively broader evidence base. A full description of the characteristics of the included studies is summarized in Table 3. An updated search was conducted on January 24, 2026, identifying no additional studies.

### MASLD diagnosis and disease characterization

Across the included studies, MASLD was diagnosed using imaging-based evidence of hepatic steatosis in addition to metabolic risk profiling, with secondary causes of liver disease excluded.^8^^,^^15^ Hepatic steatosis was commonly identified using US, MRI, or controlled attenuation parameter measures.^8^^,^^15^^,^^17^ The disease severity was primarily assessed using noninvasive fibrosis indices, such as FIB-4 score, with threshold use to classify low (<1.45) and intermediate–high (≥1.45) fibrosis risk.^21^In addition, several studies reported metabolic characteristics, including body mass index (BMI), waist-to-hip ratio, IR as measured by HOMA-IR, and lipid profiles, to characterize systemic metabolic dysfunction within MASLD cohorts.^1^While the diagnostic approaches varied across studies, they all applied integrated criteria reflecting hepatic steatosis, fibrosis burden, and metabolic abnormalities. This variability in diagnostic and staging approaches may partially account for differences in reported cognitive and neuroimaging outcomes and should be explicitly considered when interpreting results.

### Cognitive function

The 12 studies related to cognition examined the relationship between MASLD and cognitive outcomes evaluated by standardized neuropsychological tests and neuroimaging measures. Across studies, MASLD participants consistently demonstrated lower performance on cognitive assessments than control groups. The MoCA was the most frequently used screening tool, supplemented by the Digit Span Test (DST), Digit Symbol Substitution Test, Trail Making Test Parts A and B (TMT-A/B), and Mini-Mental State Examination (MMSE).^1^^,^^5^^,^^7^These findings indicate impairments in multiple cognitive domains, including attention, short-term memory, executive function, and visuospatial processing.^1^^,^^5^^,^^7^

Participants exhibited a significantly higher BMI and waist-to-hip ratios (*P* < 0.001), which were associated with lower cognitive performance on standardized tests, including the DST and MoCA, as well as increased symptoms of depression and anxiety (*P* < 0.05).^9^ IR was prevalent in this population, as reflected by the elevated HOMA-IR scores and insulin levels.^1^^,^^9^ Additionally, several studies controlled for potential confounders, including T2DM, obesity, and depressive symptoms, and found that the associations between MASLD severity and cognitive impairment largely persisted, suggesting an association with MASLD independent of measured covariates.^9^^,^^15^ Not all studies accounted for these covariates, which may contribute to variability in observed outcomes. These findings highlight the association between metabolic dysfunction, MASLD severity, and cognition. Although these metabolic factors may also influence reward-related neural circuits, here they are presented in the context of their observed associations with cognitive outcomes.

Cognitive performance varied by fibrosis stage, with individuals at more advanced stages exhibiting poorer cognitive function compared to those with mild steatosis.^9^^,^^15^ Results suggest that early diagnosis and intervention may be relevant for risk stratification of cognitive impairments.^9^ In the early stages of MASLD, mild liver pathology was not significantly associated with cognitive performance; however, in more advanced stages, poorer cognition was reflected by lower MoCA scores, which assess multiple memory domains, including working and episodic memory.^9^^,^^17^ These findings support the illness stage and progressive association between MASLD severity and cognitive impairment.

Structural neuroimaging findings revealed consistent outcomes regarding structural brain alterations in MASLD participants, including reduced cortical thickness (TH), surface area, white matter volume (WMV), and hippocampal volume.^7^^,^^17^^,^^21^ Deficits were most pronounced in visuospatial and executive function-related regions (*p* < 0.05).^6^ Liver fat content was reported to be with both hippocampal atrophy and cognitive-emotional symptoms, suggesting that subclinical structural changes occur even in cognitively normal individuals.^9^

Additionally, several studies reported vascular dysfunction in MASLD participants, including lower reactive hyperemia index, increased arterial stiffness, higher pulse wave velocity, and lower MMSE scores.^17^^,^^18^ Cerebrovascular findings included greater perfusion variability in the nucleus accumbens (NAcc) and increased white matter hyperintensities (WMH) burden, indicating compromised cerebrovascular integrity that may contribute to cognitive impairments.^16^^,^^21^

The FIB-4 index was commonly used to stratify fibrosis risk, with higher scores consistently associated with poorer cognitive outcomes and greater structural brain changes.^2^^,^^7^^,^^16^ Moderate-to-severe WMH were observed even in cognitively normal MASLD patients, highlighting early subclinical effects.^17^ Taken together, these findings suggest that metabolic dysfunction, fibrosis burden, and cerebrovascular alterations may represent interconnected processes associated with cognitive vulnerability in MASLD rather than independent correlates. While some studies did not identify a statistically significant direct association between MASLD and cognitive performance, individuals with MASLD were at increased risk for future cognitive disorders and cardiovascular disease, particularly in the presence of advanced fibrosis.^4^^,^^19^^,^^22^

### Reward system

Only two studies were included that assessed reward system effects in the context of MASLD, highlighting the limited state of research and constraining the strength of conclusions. Although other studies have reported associations between MASLD and reward function, these did not meet the eligibility criteria for this review (eg due to study design or population). While behavioral tasks and self-reported measures of anhedonia were eligible in the methods, the small number of included studies limits the generalizability of findings and underscores a notable gap in the literature regarding reward system outcomes in MASLD.

Neuroimaging findings indicated structural and functional alterations in key reward-related regions, including the NAcc, hippocampus, orbitofrontal cortex (OFC), and sensorimotor area.^1^^,^^9^ Atrophy was observed in the hippocampus and interconnected regions, reflecting disruptions within reward circuitry. ^9^ Functional connectivity (FC) analyses demonstrated reduced coupling between the right hippocampus and OFC, suggesting impaired communication across reward networks in participants with advanced liver disease.^9^ Using whole-brain connectivity mapping with a right hippocampal seed-based approach, which demonstrated reduced FC between the right hippocampus and right OFC, suggesting disrupted communication within reward circuits and potential vulnerability to metabolic and structural perturbations in MASLD.^9^

A higher NAcc scaled coefficient of variation (sCoV) was reported, independent of age, sex, BMI, diabetes, and hypertension.^17^ Increased NAcc sCoV indicates greater variability in cerebral blood flow (CBF) and reduced vascular efficiency, a pattern commonly seen in early stages of cardiovascular disease and metabolic dysfunction.^17^ A linear regression analysis examined plasma inflammatory markers (cytokines, acute-phase proteins, and complement factors), revealing that elevated plasma concentrations of interleukin-6 (IL-6) and tumor necrosis factor alpha (TNF-*α*), pro-inflammatory cytokines induced by upstream mediators such as IL-1 beta (IL-1β), were significantly associated with higher NAcc sCoV across all models, linking systemic inflammation to functional impairment within reward circuitry.^17^ These findings suggest that MASLD-related metabolic and inflammatory disturbances are associated with alterations in neural substrates underlying reward processing, motivation, and affective regulation.

Advanced brain MRI techniques incorporating novel biomarkers of grey and white matter integrity and cerebrovascular health further provides preliminary support for a neurobiological basis.^9^^,^^17^ Imaging findings revealed stronger links between structural and functional alterations in reward-related regions and MASLD severity than blood-based markers alone. ^9^^,^^17^ Specifically, later stages of MASLD were associated with reduced WMV, impaired blood perfusion, and poorer cerebrovascular health, indicating progressive disruptions of neural substrates underlying reward processing.^9^^,^^15^^,^^17^ Collectively, these findings suggest that metabolic, inflammatory, and cerebrovascular alterations in MASLD may converge on frontostriatal and limbic networks involved in motivation and reward processing, although the limited number of studies necessitates cautious interpretation and these findings should be considered preliminary. Overall, while the current evidence is limited to neuroimaging outcomes, it suggests a possible association between MASLD and disruptions in reward circuits, underscoring the need for future studies using behavioral and self-report measures to fully characterize reward-related dysfunction.

Across studies, findings were generally consistent in direction, with MASLD associated with poorer performance across multiple cognitive domains, although effect sizes varied.

### Methodological quality

Table 1 presents the risk-of-bias and quality assessment for the included cohort and cross-sectional studies. Overall, these studies demonstrated variable methodological quality. Common strengths included clearly stated research objectives, well-defined study populations, and the use of valid and reliable outcome measures. However, several studies exhibited methodological limitations, most frequently related to inadequate control of confounding variables, lack of blinded assessors to the exposed status, and limited reporting on the validity or consistency of exposure measurement. Additionally, cross-sectional studies were limited by the inability to assess both exposure and outcomes during the study period. As a result, cohort and cross-sectional studies were classified into good, fair, and poor quality categories based on the number and severity of these limitations.

**Table 1.** Risk of Bias/Quality Assessment of the Included Studies Using the NIH Quality Assessment of Cohort and Cross-Sectional Studies Table 1. long description.

							Item								
Study	1	2	3	4	5	6	7	8	9	10	11	12	13	14	Quality Rating
Celikbilek et al.^14^	✓	✓	NR	✓	✓	✓	✓	✓	✓	X	X	✓	NR	✓	Good
Jang et al.^8^	✓	✓	✓	✓	✓	✓	✓	✓	✓	X	X	✓	X	X	Fair
Jin et al.^1^	✓	✓	NR	✓	X	✓	✓	✓	✓	X	X	X	X	X	Fair
Xu et al.^21^	✓	✓	NR	✓	X	✓	✓	✓	✓	X	X	X	X	X	Fair
Weinstein et al.^1^ ^9^	✓	✓	✓	✓	X	✓	✓	✓	✓	X	X	X	X	✓	Fair
Seidel et al.^17^	✓	✓	NR	✓	✓	✓	✓	✓	✓	✓	✓	X	X	X	Fair
Miao et al.^7^	✓	✓	✓	✓	X	✓	X	✓	✓	X	X	X	X	X	Poor
Wu et al.^9^	✓	✓	NR	✓	X	✓	✓	✓	✓	X	X	✓	X	X	Fair
Parikh et al.^5^	✓	✓	✓	✓	✓	✓	✓	✓	✓	✓	X	X	NR	X	Fair
Xiao et al.^20^	✓	✓	✓	✓	✓	✓	X	✓	✓	X	✓	X	✓	✓	Good
Yilmaz et al.^22^	✓	✓	✓	✓	X	✓	✓	✓	✓	✓	✓	X	✓	✓	Good
Li et al.^15^	✓	✓	✓	✓	✓	✓	✓	X	✓	X	X	X	NR	✓	Fair
Lin et al.^15^	✓	✓	X	✓	✓	✓	CD	✓	✓	X	X	X	NR	✓	Fair

*Note:* Quality was assessed using the National Institutes of Health (NIH) Quality Assessment Tool for Observational Cohort and Cross-Sectional Studies. Each study was rated across tool-specific items and assigned an overall quality rating (good, fair, or poor) based on risk-of-bias, confounding control, and methodological rigor. Abbreviations: CD, Cannot Determine; NR, Not Reported.

Table 2 summarizes the methodological quality of the included case–control studies. While there was a lack of case–control studies reviewed, the single study clearly defined cases and controls and applied appropriate outcome definitions; however, the methodological quality varied with respect to exposure assessment and comparability between groups. Common sources of potential bias included insufficient justification for sample size, nonblinded assessors to the case or control exposure status, and an incomplete adjustment for key confounders. These issues contributed to variability in the overall quality of the article, categorizing it as fair according to the NIH criteria.^13^ Across all study designs, methodological quality was considered when interpreting the findings but was not used as an exclusion criterion. Accordingly, variability in study quality and incomplete adjustment for metabolic and vascular confounders should be considered when interpreting the magnitude and consistency of the reported associations between MASLD, cognitive outcomes, and reward-related findings.

**Table 2.** Risk of Bias/Quality Assessment of the Included Studies Using the NIH Quality Assessment of Case–Control Studies Table 2. long description.

							Item						
Study	1	2	3	4	5	6	7	8	9	10	11	12	Quality Rating
Tuttolomondo et al.^18^	✓	✓	X	✓	✓	✓	X	CD	✓	✓	X	X	Fair

*Note:* Quality was assessed using the National Institutes of Health (NIH) Quality Assessment Tool for Case–Control Studies. Studies were evaluated across NIH criteria and assigned an overall rating (good, fair, or poor) based on methodological quality, confounding control, and risk-of-bias. Abbreviations: CD, Cannot Determine.

Table 3 presents the characteristics of the included studies, including the first author and year of publication, study design, population characteristics, and sample size. It further details the methods used to assess cognitive functioning, the measures used to evaluate reward-related processes, and the diagnostic methods used to determine fatty liver status. Variability in study design and measurement approaches is evident across studies, which may influence the differences in the reported findings.

**Table 3.** Characteristics of Included Studies Table 3. long description.

Study	Design	Population	Sample size	MASLD diagnosis/severity	Diagnostic modality	Cognitive domains assessed	Cognitive measures	Reward measures	Key neuro/brain measures	Effect size (CI: 95%), *p*-value
Celikbilek et al.^14^	Prospective Cross- Sectional	Bozok University School of Medicine Yozgat	143	MASLD (Steatosis)	AU	Global cognition	Turkish MoCA	N/A	N/A	MoCA: *p* < 0.001; AU: *p* = NR
Jang et al.^8^	Cross-Sectional	Samsung Hospital Screening Center	1260	MASLD (Fibrosis risk stratification)	MRI, FIB–4, NFS	Global cognition	MMSE	N/A	N/A	WMH (FIB–4): OR 1.14 (0.72–1.82); OR 1.77 (1.13–2.78); *p* = 0.016 WMH (NFS): OR 0.92 (0.53–1.59); OR 2.05 (1.34–3.14); *p* = 0.002
Jin et al.^1^	Cross-Sectional	Hangzhou Normal University Affiliated Hospital	136	MASLD (steatosis/fibrosis)	MRI-PDFF, CAP	Executive function, attention, memory	HAD, MoCA, DST, TMT-A/B	N/A	N/A	CAP: *p* > 0.05; DST: *p* < 0.001; HAD: *p* = 0.002; MoCA: *p* < 0.001; MRI-PDFF: *p* = 0.002; TMT-A: *p* > 0.05; TMT-B: *p* = 0.002
Li et al.^15^	Mendelian Randomization	UK Biobank, SSGAC	108 258	Genetic MASLD proxy	CT, MRI, biopsy (GWAS-defined exposure)	Attention (processing speed)	Derived by GWAS, COGENT	N/A	N/A	VD: OR (MASLD) 1.66 (1.08–2.55), *p* = 0.020; VD: OR (fibrosis) 1.85 (1.17–2.92), *p* = 0.009
Lin et al.^16^	Mendelian Randomization	FinnGens GWAS	342 499	Genetic MASLD proxy	NAS Score	Global cognition	MoCA	N/A	N/A	NAS: *p* = 0.008–0.048; MoCA: *p* = NR
Miao et al.^7^	Cross-Sectional	Drum Tower Hospital Nanjing	320	MASLD (steatosis/fibrosis)	NAS, LI	Global cognition	MMSE, MoCA, RBANS	N/A	N/A	MMSE: *p* < 0.001; MMSE: *p* = 0.161; MoCA: *p* = 0.118; RBANS: *p* = 0.084; NAS: *p* = 0.027 to 0.047; LI: *p* = NR
Parikh et al.^5^	Retrospective Cohort	UK Biobank (40–69 y)	455 426	MASLD risk via fibrosis index	FIB–4	Attention/executive function	DDST, TMT-A/B, DSST	N/A	N/A	*β* (DSST) = −0.46 (95% CI −0.66 to −0.26, *p* < 0.001); *β* (TMT-A) = 0.02 (95% CI 0.01–0.04, *p* = 0.002); *β* (TMT-B) = 0.03 (95% CI 0.01–0.04, *p* < 0.001); *β* (FIB–4) = −3768.0 (95% CI −5278.8 to −2257.2, *p* < 0.001)
Seidel et al.^17^	Cross-Sectional	Rijnstate Hospital (BMI ≥30, 45–55 y)	70	MASLD (fibrosis risk)	NFS	Executive function, attention	MoCA, TAP, COWAT	NAcc-sCoV, WMH count	WMH count	*β* (Nacc-sCoV) = 0.295, *p* = 0.001; TAP: *p* = NR; MoCA: *p* = NR; NFS: *p* = NR
Tuttolomondo et al.^18^	Case–Control	Palermo University Hospital	209	MASLD (fibrosis staging)	LSM (>6 kPa)	Global cognition	MMSE	N/A	N/A	MMSE: *p* = 0.005; LSM: *p* = 0.005
Weinstein et al.^19^	Cross-Sectional	FHS cohorts	1287	MASLD (imaging-based steatosis)	MDCT, NFS	Executive function, memory, visuospatial	WMS–5, TMT-A/B, SIM, HVOT	N/A	N/A	*β* (TMT-A) = −0.11 ± 0.05, *p* = 0.028; *β* (TMT-B) = −0.02 ± 0.03, *p* = 0.375; *β* (SIM) = −2.22 ± 0.07, *p* = 0.009; *β* (HVOT) = −0.01 ± 0.14, *p* = 0.971
Wu et al.^9^	Retrospective Cohort	Hangzhou Normal University Affiliated Hospital (18–60 y)	136	MASLD (steatosis)	MRI-PDFF	Global cognition, executive function	MoCA, DST, TMT-A/B, HAD	Seed-based FC analysis	Functional connectivity (hippocampus-OFC)	MRI-PDFF: *p* = NR; MoCA: *p* < 0.001; DST: *p* = 0.047; TMT-A: *p* = 0.656; TMT-B: *p* = 0.043; HAD: *p* < 0.001
Xiao et al.^20^	Prospective Cohort	Rotterdam Ommoord (≥45 y)	3975 (Set 1), 24 577 (Set 2), 3300 (Set 3)	MASLD (steatosis/fibrosis risk strata)	US, FLI ≥60, LSM ≥8.0 kPa	Psychiatric + cognitive screening	DSM-III-R	N/A	N/A	HR (FLI) = 0.92 (95% CI 0.69–1.22); HR (LSM) = 1.01 (95% CI 0.92–1.10); HR (US) = 0.84 (95% CI 0.61–1.16)
Xu et al.^21^	Cross-Sectional	Hangzhou Normal University Affiliated Hospital	64	MASLD (steatosis)	MRI-PDFF	Attention, executive function	MoCA, DST, TMT-A	N/A	N/A	MRI-PDFF: *p* = 0.405; MoCA: *p* = 0.000; DST: *p* = 0.000; TMT A: *p* = 0.023; HOMA-IR: *p* = 0.007

*Note:* MASLD was defined using heterogeneous approaches including imaging (US, MRI, MRI-PDFF, CT, transient elastography), fibrosis scores (FIB-4, NAFLD fibrosis score, fatty liver index), or genetic proxies in Mendelian randomization studies. Cognitive outcomes were grouped post hoc into global cognition, executive function, attention/processing speed, and memory domains. Reward-related outcomes included behavioral, self-report, and neuroimaging-based measures of reward circuitry. Neuroimaging findings included structural, functional connectivity, and cerebrovascular markers within frontostriatal and limbic networks. Due to methodological heterogeneity, effect estimates were not pooled and results were synthesized qualitatively. *β* (B) = regression coefficient; OR = odds ratio; HR = hazard ratio; CI = confidence interval; *p* values <0.05 denote statistically significant.

Abbreviations: 15-WLT, 15-Word Learning Test; AU, abdominal ultrasound; *β*, beta coefficient; CAP, controlled attenuation parameter; CI, confidence interval; COGENT, Controlled Oral Word Association Test; COWAT, Controlled Oral Word Association Test; CT, computed tomography; DSM-III-R, Diagnostic and Statistical Manual of Mental Disorders, third edition, revised; DSST, Digit Symbol Substitution Test; FC, functional connectivity; FHS, Framingham Heart Study; FIB-4, fibrosis-4 index; FLI, fatty liver index; GWAS, genome-wide association study; HAD, Hospital Anxiety and Depression Scale; HOMA-IR, Homeostatic Model Assessment of Insulin Resistance; HR, hazard ratio; HVOT, Hooper Visual Organization Test; LDST, Letter Digit Substitution Test; LI, liver index; LSM, liver stiffness measurement; MASLD, metabolic dysfunction-associated steatotic liver disease; MDCT, multidetector computed tomography; MMSE, Mini-Mental State Examination; MoCA, Montreal Cognitive Assessment; MRI, magnetic resonance imaging; MRI-PDFF, magnetic resonance imaging–proton density fat fraction; NAS, NAFLD activity score; NAcc-sCoV, Nucleus Accumbens Scaled Coefficient of Variation; NFS, NAFLD fibrosis score; NR, not reported; OFC, orbitofrontal cortex; OR, odds ratio; RBANS, Repeatable Battery for the Assessment of Neuropsychological Status; SIM, Similarities Subtest; SSGAC, Social Science Genetic Association Consortium TAP, Test of Attentional Performance; TE, transient elastography; TMT-A/B, Trail Making Test, Parts A and B; UK Biobank, United Kingdom Biobank; US, ultrasound; VD, vascular dementia; WFT, Word Fluency Test; WMH, white matter hyperintensities; WMS-5, Wechsler Memory Scale, fifth edition.

## Discussion

This review synthesizes the extant evidence reporting on the association between MASLD and brain functioning, with a particular focus on cognitive performance and reward-related processing. Across the 12 cognition-focused studies and the two reward-related studies, the findings consistently suggest that MASLD is associated with poorer cognitive performance, structural brain alterations, and early dysfunction in reward-related neural circuitry. Collectively, the evidence also suggests a stronger association between brain abnormalities and greater severity of liver fibrosis. Taken together, these findings suggest that metabolic, inflammatory, and vascular processes associated with MASLD may converge on neural systems that support executive and motivational functions, supporting a plausible framework linking liver disease severity with neurocognitive vulnerability.

Compared with controls, individuals with MASLD demonstrated significantly lower performance on several cognitive measures, including the MoCA, DST, and TMT-A/B.^1^^,^^9^ Deficits were most evident in executive function, attention, and working memory.^10^^,^^17^ Notably, fibrosis burden, frequently indexed using the FIB-4 score, was consistently associated with worse cognitive performance across studies, supporting a potential dose–response relationship between liver pathology and cognitive dysfunction.^7^ Converging neuroimaging findings further indicated reduced cortical thickness, hippocampal volume, WMV, and increased WMH, including among MASLD participants without overt cognitive impairment.^5^^,^^17^ The foregoing results suggest that subclinical brain changes may occur early in the disease course and may be more pronounced with increasing fibrosis severity.

Separately, we were only able to identify two studies directly examining reward system functioning in MASLD. Available evidence points to alterations in key reward-related regions, including the hippocampus, NAcc, and OFC.^9^^,^^17^ Reported abnormalities included altered CBF within the NAcc and reduced functional connectivity between the hippocampus and OFC, even in the early stages of the disease.^9^ The aforementioned neural changes were reported alongside markers of systemic inflammation, IR, abdominal obesity, and depressive symptoms, implicating plausible mechanistic pathways linking hepatic dysfunction to motivational and affective disturbances.^9^^,^^17^ Taken together, the foregoing findings suggest that MASLD is not solely a metabolic–hepatic condition but a multisystem disorder with clinically meaningful neurocognitive and neuropsychiatric consequences. However, given that only two studies directly examined reward system functioning, these results should be interpreted cautiously and considered preliminary; alternative explanations, including shared effects of obesity and IR on reward processing, cannot be excluded. Collectively, the available evidence is consistent with a framework in which MASLD-related metabolic abnormalities are associated with alterations in reward processing and executive control, potentially reflecting early cognitive inefficiency and vulnerability to neuropsychiatric symptoms.

The following mechanisms are theoretical interpretations informed by broader literature and are not directly established by the included studies. In addition to these systemic and cerebrovascular mechanisms, emerging evidence further implicates neuroinflammation as a key pathway linking MASLD to brain dysfunction. Chronic hepatic inflammation and metabolic dysregulation promote the release of pro-inflammatory cytokines (eg TNF-*α*, IL-6, IL-1*β*) and IR, which may compromise the blood–brain barrier integrity and facilitate central immune activation, including microglial activation, oxidative stress, and mitochondrial dysfunction.^23^^–^^28^ These processes have been linked to neuronal injury within regions critical for cognition and reward processing, such as the hippocampus, prefrontal cortex, and dopaminergic pathways, and may underlie impairments in executive function, memory, and motivation.^23^^–^^28^ While these mechanisms are biologically plausible and supported by broader literature, they remain inferential within the context of the included studies and should be interpreted cautiously. Emerging treatments for MASLD, including GLP-1 receptor agonists (eg semaglutide), may offer neuroprotective effects by attenuating neuroinflammation and oxidative stress, while agents such as resmetirom may indirectly reduce neuroinflammatory burden through improvements in hepatic and systemic metabolic function.^23^^–^^28^

Despite these mechanistic insights, the directionality of the observed relationships between MASLD and neurocognitive and reward-related alterations remains uncertain. The predominance of cross-sectional evidence in the included studies precludes conclusions regarding temporality or causality. Several nonmutually exclusive explanatory models may account for the observed associations. MASLD-related metabolic, inflammatory, and vascular disturbances may directly contribute to neurocognitive dysfunction. In addition, preexisting cognitive or behavioral vulnerabilities may increase the risk of MASLD through the lack of adherence to healthy lifestyle behaviors and impaired decision-making. Both MASLD and neurocognitive alterations may arise from shared upstream pathways, including systemic IR, chronic inflammation, and broader cardiometabolic risk factors.

An important and equally plausible interpretation is that MASLD may function as a marker of systemic metabolic dysfunction rather than a direct driver of neurocognitive changes. Shared factors such as IR, inflammation, and adiposity may underlie both liver disease and brain alterations. Reverse causation should also be considered, as impairments in executive function and motivation may contribute to poorer lifestyle behaviors that increase the risk of MASLD. Given the predominance of cross-sectional evidence, these findings should be interpreted cautiously, and longitudinal studies are needed to clarify directionality and causality.

A related consideration is the role of residual confounding, which remains a significant limitation across the included studies. Variables in this relationship may function as confounders, mediators, or colliders. IR, visceral adiposity, and socioeconomic factors may act as confounders influencing both MASLD and cognitive outcomes, whereas systemic inflammation and cerebrovascular dysfunction may lie on the causal pathways as mediators. Conditioning on variables such as clinical diagnosis or healthcare utilization may also introduce collider bias, potentially distorting observed associations. Although some studies adjust for T2DM and BMI, these measures are incomplete proxies for metabolic dysfunction. Residual confounding from metabolic, behavioral, and socioeconomic factors, as well as cognitive reserve (eg education level) and subthreshold psychiatric symptoms, may independently influence cognitive performance and are not consistently accounted for. Accordingly, the observed association may reflect shared risk pathways rather than liver-specific effects.

Our findings have several implications for clinical care and population health. First, the evidence suggests the inclusion of cognitive screening into the routine assessment of patients with MASLD. Cognitive deficits in MASLD, even at subtle levels, may reduce patients’ ability to understand, prepare, and adhere to lifestyle or pharmacological interventions, making early identification clinically actionable. While no specific screening tool for cognitive function has been identified as valid, reliable, user-friendly, at no cost, integrated with electronic health records and able to detect presence and change in cognitive function in persons specifically living with MASLD. Commonly used screening tools, such as the MoCA, are practical for identifying major neurocognitive impairment but may lack sensitivity to detect subtle or early domain-specific cognitive changes observed in MASLD. In addition, it is amply documented that subjective and objective cognitive impairments often do not correlate as a cross-sectional measure. Cognitive measurement tools that have been specifically validated and could be of potential utility in the psychiatric population include the THINC-it tool, the Perceived Deficit Questionnaire-Five Item, and/or the Screen for Cognitive Impairment in Psychiatry.^10^^,^^29^^–^^33^Second, preliminary evidence of reward-circuit involvement provides a neurobiological basis for the elevated burden of depression, anhedonia, and motivational impairments observed in MASLD populations.^2^ Such deficits in motivation and reward processing are clinically actionable because they may directly reduce engagement with lifestyle and medical treatments, thereby contributing to a self-perpetuating cycle of worsening metabolic and hepatic disease progression. Recognition of these cognitive and motivational changes may therefore have practical implications for treatment adherence, lifestyle modification, and long-term disease management in MASLD.

The consistent associations between liver fibrosis severity, systemic inflammation, IR, cerebrovascular dysfunction, and brain alterations underscore the importance of early identification and prevention from a public health perspective.^34^Enhancing screening for MASLD and improving early detection of fibrosis severity represent critical preliminary steps in mitigating downstream neurocognitive consequences. Early identification may facilitate timely risk stratification and the implementation of preventive strategies before overt cognitive impairment emerges.^9^ An alternative interpretation is that MASLD may function as a clinical marker of systemic metabolic dysfunction rather than a direct driver of neurocognitive changes, further underscoring the importance of addressing shared cardiometabolic risk factors.

Given the robust association between metabolic dysfunction and cognition, cost-effective and accessible interventions should be prioritized. Lifestyle and behavioral modifications targeting IR, hepatic accumulation, and vascular health, including dietary changes, physical activity, and weight management, may offer dual benefits for liver and brain function.^23^^–^^28^ Additionally, emerging pharmacological treatments for MASLD, including incretin-based therapies (eg GLP-1 receptor agonists), may provide improvement in cognitive and reward-related outcomes through their effects on metabolic and brain insulin signaling.^23^^–^^28^ Emerging evidence suggests brain IR represents a potential therapeutic target for cognitive impairment and anhedonia, highlighting the potential relevance of such treatments beyond hepatic outcomes.^28^

Finally, the results highlight that MASLD is a disorder characterized by systemic metabolic dysregulation with meaningful implications for brain function, particularly within neural circuits supporting cognition and reward. Instead of implying a nonspecific bidirectional liver–brain interaction, the available evidence suggests a mechanistic framework in which MASLD-related metabolic abnormalities, such as IR, inflammation, and dyslipidemia, may influence reward processing and executive control before the onset of overt neuropsychiatric disease. An alteration in motivation, effort valuation, and decision-making may represent early behavioral manifestations of metabolic dysfunction, with downstream consequences for both cognitive performance and psychiatric vulnerability.

The emerging behavioral and clinical evidence is consistent, demonstrating that peripheral metabolic factors independently modulate reward-related processes. In published articles, metabolic dysregulation is shown to alter effort-based decision-making in major depressive disorder, supporting a direct link between metabolic state and reward-related behavior.^26^ Similarly, another study suggests that obesity provides converging evidence that IR, dyslipidemia, and adiposity are associated with disruptions in reward learning, effort valuation, and cost–benefit decision-making, even after controlling for mood symptoms.^25^ Ultimately, these findings suggest that MASLD may confer risk for cognitive and psychiatric comorbidities through shared metabolic–behavioral pathways that precede, and potentially contribute to, the development of clinical neuropsychiatric outcomes.

Several limitations should be considered when interpreting these findings. First, most of the included studies used cross-sectional designs, limiting the ability to infer causality or temporal relationships between MASLD and cognitive or reward-related impairments. To further support these findings, longitudinal studies are needed to determine whether cognitive and neural alterations result from liver disease progression. Second, there was substantial heterogeneity in diagnostic criteria for MASLD, fibrosis staging, neurocognitive and neuroimaging assessments, and covariate adjustment strategies, limiting comparability across studies. Given this methodological variability and differences in reported outcomes, a quantitative meta-analysis was not feasible, and findings were synthesized qualitatively. Third, only two studies directly examined reward system functioning, highlighting a significant gap in the literature. As a result, conclusions regarding reward-related processes should be interpreted cautiously. Additionally, many studies relied on markers such as FIB-4 rather than liver biopsy, which may underestimate disease severity. Potential confounders, including lifestyle factors, socioeconomic status, and subclinical psychiatric symptoms, were not consistently controlled across studies, which may partially account for the observed associations. The exclusion of participants with overt neurological or psychiatric disorders, while methodologically necessary, may limit the generalizability of findings to real-world MASLD populations, in whom such comorbidities are common. Furthermore, incomplete adjustment for metabolic and vascular comorbidities common in MASLD populations (eg T2DM and obesity) limits the ability to determine the independent contribution of liver pathology to observed cognitive and structural brain changes, reinforcing the need for cautious interpretation of causality.

Interpretation of these findings should take into account the overall methodological quality of the included studies. While several studies demonstrated robust outcome assessment and clearly defined study populations, many were limited by cross-sectional designs and incomplete control of confounding. As a result, the observed associations should be interpreted as correlational rather than causal, highlighting the need for studies capable of disentangling liver-specific effects from broader metabolic and vascular risk factors.

Future research should prioritize longitudinal, mechanistic studies that integrate liver imaging, inflammatory biomarkers, and behavioral assessments of reward processing. Additionally, expanding the literature of reward system dysfunction may be particularly valuable for understanding motivational deficits and psychiatric comorbidities in MASLD. Refining these studies will be essential for developing targeted interventions aimed at mitigating cognitive decline and improving the quality of life in individuals with MASLD. Clinicians are encouraged to screen for persons with MASLD for serious mental illness. Along with risk factor modification and lifestyle change, the Food and Drug Administration has approved two therapeutics in the treatment of persons with MASLD whose condition has advanced to include fibrosis and inflammation (ie resmetirom, semaglutide).^23^^,^^25^ In addition to their efficacy in the treatment of MASLD, emerging evidence also suggests that GLP-1 RAs may target mechanisms relevant to cognition and reward, providing the impetus for studies to evaluate their potential treatment and preventative effects in persons with substance use, alcohol use, depressive, and bipolar disorders.^23^^,^^27^^,^^28^ Future research should specifically focus on the extent to which MASLD induces impairment in cognitive function and reward is occurring with people with SMI. In addition, it would be informative to the field to determine the extent does the presence of MASLD account for deficits observed in cognitive and reward function in persons living with MASLD.^25^^,^^35^^,^^36^ Clarifying whether MASLD independently accounts for a proportion of cognitive and motivational deficits observed in metabolically vulnerable populations would represent an important step toward precision-based prevention and treatment strategies.

## Figure 1. Long description

At the top, a brown header reads Identification of Studies via Databases. The process starts with Records identified from asterisk Databases n equals 206. A rightward arrow leads to Records removed before screening Duplicate records removed n equals 38. A downward arrow leads to Records screened n equals 168. Rightward, Records excluded double asterisk n equals 121. Downward, Reports sought for retrieval n equals 47. Rightward, Reports not retrieved n equals 17. Downward, Reports assessed for eligibility n equals 30. Rightward, Reports excluded Population Conflict n equals 4, Study Design n equals 7, Objective n equals 5. Downward, Studies included in review n equals 14. The leftmost column labels the stages vertically: Identification, Screening, Included.

Navigate back to Figure 1..

## Table 1. Long description

The table has 16 columns. The first column lists study names, followed by columns labeled 1 to 14 representing N I H assessment items, and the last column shows the overall quality rating. For each study, check marks indicate criteria met, X indicates not met, NR means not reported, and CD means cannot determine. Celikbilek et al. is rated good, meeting most criteria except items 3, 10, 11, and 13 (NR or X). Jang et al. is rated fair, with X for items 10, 11, 13, and 14. Jin et al., Xu et al., Weinstein et al., Seidel et al., Wu et al., Parikh et al., Li et al., and Lin et al. are all rated fair, each missing several criteria, especially in items 5, 10 to 14. Miao et al. is rated poor, missing items 5, 7, 10 to 14. Xiao et al. and Yilmaz et al. are rated good, meeting most criteria except for a few X or NR. Lin et al. has CD for item 7. The table footnote explains that quality was assessed using the N I H tool, with ratings based on risk-of-bias, confounding control, and methodological rigor. Abbreviations: CD is cannot determine, NR is not reported.

Navigate back to Table 1..

## Table 2. Long description

The table has 14 columns. The first row lists headers: Study, Items 1 to 12, and Quality Rating. The second row contains the study ‘Tuttolomondo et al.’ with the following ratings for each item: Item 1, check mark; Item 2, check mark; Item 3, X; Item 4, check mark; Item 5, check mark; Item 6, check mark; Item 7, X; Item 8, CD; Item 9, check mark; Item 10, check mark; Item 11, X; Item 12, X. The final column assigns the study a Quality Rating of Fair. The note below the table states that quality was assessed using the National Institutes of Health Quality Assessment Tool for Case-Control Studies, with ratings based on methodological quality, confounding control, and risk-of-bias. Abbreviations: CD means Cannot Determine.

Navigate back to Table 2..

## Table 3. Long description

The table contains 12 columns: Study, Design, Population, Sample size, MASLD diagnosis/severity, Diagnostic modality, Cognitive domains assessed, Cognitive measures, Reward measures, Key neuro/brain measures, and Effect size with confidence interval and p-value. From the top row, column headers are as listed. Each subsequent row details a study: Celikbilek et al. used a prospective cross-sectional design at Bozok University with 143 participants, MASLD steatosis diagnosed by abdominal ultrasound, assessing global cognition via Turkish MoCA, with MoCA p less than 0.001. Jang et al. conducted a cross-sectional study at Samsung Hospital with 1260 participants, MASLD fibrosis risk stratified by MRI, FIB-4, and NFS, global cognition assessed by MMSE, with WMH (FIB-4) odds ratio 1.14 (0.72–1.82), 1.77 (1.13–2.78), p equals 0.016; WMH (NFS) odds ratio 0.92 (0.53–1.59), 2.05 (1.34–3.14), p equals 0.002. Jin et al. performed a cross-sectional study at Hangzhou Normal University with 136 participants, MASLD steatosis/fibrosis by MRI-PDFF and CAP, executive function, attention, and memory assessed by HAD, MoCA, DST, TMT-A/B, with DST p less than 0.001, HAD p equals 0.002, MoCA p less than 0.001, MRI-PDFF p equals 0.002, TMT-A p greater than 0.05, TMT-B p equals 0.002. Li et al. used Mendelian randomization with UK Biobank and SSGAC, 108258 participants, genetic MASLD proxy, diagnostic modalities CT, MRI, biopsy, attention assessed by GWAS and COGENT, vascular dementia odds ratio (MASLD) 1.66 (1.08–2.55), p equals 0.020; (fibrosis) 1.85 (1.17–2.92), p equals 0.009. Lin et al. used Mendelian randomization with FinnGens GWAS, 342499 participants, genetic MASLD proxy, NAS score, global cognition by MoCA, NAS p equals 0.008–0.048. Miao et al. conducted a cross-sectional study at Drum Tower Hospital with 320 participants, MASLD steatosis/fibrosis by NAS and LI, global cognition by MMSE, MoCA, RBANS, MMSE p less than 0.001 and p equals 0.161, MoCA p equals 0.118, RBANS p equals 0.084, NAS p equals 0.027 to 0.047. Parikh et al. performed a retrospective cohort study in UK Biobank (40–69 years), 455426 participants, MASLD risk via FIB-4, attention/executive function by DDST, TMT-A/B, DSST, beta (DSST) equals minus 0.46 (95 percent CI minus 0.66 to minus 0.26, p less than 0.001), beta (TMT-A) equals 0.02 (0.01–0.04, p equals 0.002), beta (TMT-B) equals 0.03 (0.01–0.04, p less than 0.001), beta (FIB-4) equals minus 3768.0 (minus 5278.8 to minus 2257.2, p less than 0.001). Seidel et al. used a cross-sectional design at Rijnstate Hospital (BMI at least 30, 45–55 years), 70 participants, MASLD fibrosis risk by NFS, executive function and attention by MoCA, TAP, COWAT, NAcc-sCoV, WMH count, beta (Nacc-sCoV) equals 0.295, p equals 0.001. Tuttolomondo et al. conducted a case-control study at Palermo University Hospital, 209 participants, MASLD fibrosis staging by LSM (greater than 6 kPa), global cognition by MMSE, MMSE p equals 0.005, LSM p equals 0.005. Weinstein et al. performed a cross-sectional study in FHS cohorts, 1287 participants, MASLD imaging-based steatosis by MDCT, NFS, executive function, memory, visuospatial by WMS-5, TMT-A/B, SIM, HVOT, beta (TMT-A) equals minus 0.11 plus or minus 0.05, p equals 0.028; beta (TMT-B) equals minus 0.02 plus or minus 0.03, p equals 0.375; beta (SIM) equals minus 2.22 plus or minus 0.07, p equals 0.009; beta (HVOT) equals minus 0.01 plus or minus 0.14, p equals 0.971. Wu et al. used a retrospective cohort at Hangzhou Normal University (18–60 years), 136 participants, MASLD steatosis by MRI-PDFF, global cognition and executive function by MoCA, DST, TMT-A/B, HAD, seed-based FC analysis, functional connectivity (hippocampus-OFC), MoCA p less than 0.001, DST p equals 0.047, TMT-A p equals 0.656, TMT-B p equals 0.043, HAD p less than 0.001. Xiao et al. conducted a prospective cohort in Rotterdam Ommoord (at least 45 years), 3975 (Set 1), 24577 (Set 2), 3300 (Set 3), MASLD steatosis/fibrosis risk by US, FLI at least 60, LSM at least 8.0 kPa, psychiatric and cognitive screening by DSM-III-R, HR (FLI) equals 0.92 (95 percent CI 0.69–1.22), HR (LSM) equals 1.01 (0.92–1.10), HR (US) equals 0.84 (0.61–1.16). Xu et al. performed a cross-sectional study at Hangzhou Normal University, 64 participants, MASLD steatosis by MRI-PDFF, attention and executive function by MoCA, DST, TMT-A, MRI-PDFF p equals 0.405, MoCA p equals 0.000, DST p equals 0.000, TMT A p equals 0.023, HOMA-IR p equals 0.007. Abbreviations and statistical notes are provided below the table.

Navigate back to Table 3..
